# Comprehensive Profiling of EBV Gene Expression and Promoter Methylation Reveals Latency II Viral Infection and Sporadic Abortive Lytic Activation in Peripheral T-Cell Lymphomas

**DOI:** 10.3390/v15020423

**Published:** 2023-02-02

**Authors:** Joanna W. Y. Ho, Lili Li, Kai Yau Wong, Gopesh Srivastava, Qian Tao

**Affiliations:** 1 Department of Pathology, The University of Hong Kong, Hong Kong; 2 School of Biomedical Sciences, LKS Faculty of Medicine, The University of Hong Kong, Hong Kong; 3 Cancer Epigenetics Laboratory, Department of Clinical Oncology, State Key Laboratory of Translational Oncology, Sir YK Pao Center for Cancer and Li Ka Shing Institute of Health Sciences, The Chinese University of Hong Kong, Hong Kong; 4 Johns Hopkins Singapore, Singapore

**Keywords:** peripheral T-cell lymphoma, EBV, latency, LMP1, Zta, promoter, CpG methylation

## Abstract

Epstein-Barr virus (EBV) latency patterns are well defined in EBV-associated epithelial, NK/T-cell, and B-cell malignancies, with links between latency stage and tumorigenesis deciphered in various studies. *In vitro* studies suggest that the oncogenic activity of EBV in T-cells might be somewhat different from that in EBV-tropic B lymphoid cells, prompting us to study this much less investigated viral gene expression pattern and its regulation in nine EBV+ peripheral T-cell lymphoma (PTCL) biopsies. Using frozen specimens, RT-PCR showed 6/7 cases with a latency II pattern of EBV gene expression. Analyses of EBNA1 promoter usage and CpG methylation status in these six cases showed that only Qp was used, while Cp, Wp, and Fp were all silent. However, the remaining case showed an exceptionally unique latency III type with lytic activation, as evidenced by EBV lytic clonality and confirmed by the full usage of Cp and Qp as well as weakly lytic Fp and Wp, fully unmethylated Cp and marginally unmethylated Wp. Further immunostaining of the eight cases revealed a few focally clustered LMP1+ cells in 7/8 cases, with rare isolated LMP1+ cells detected in another case. Double immunostaining confirmed that the LMP1+ cells were of the T-cell phenotype (CD3+). In 6/8 cases, sporadically scattered Zta+ cells were detected. Double staining of EBER-ISH with T-cell (CD45RO/UCHL1) or B-cell (CD20) markers confirmed that the vast majority of EBER+ cells were of the T-cell phenotype. Predominant type-A EBV variant and LMP1 30-bp deletion variant were present, with both F and f variants detected. In summary, the EBV gene expression pattern in PTCL was found to be mainly of latency II (BART+EBNA1(Qp)+LMP1+LMP2A+BZLF1+), similar to that previously reported in EBV-infected nasopharyngeal epithelial, NK/T-cell, and Hodgkin malignancies; however, fully lytic infection could also be detected in occasional cases. Rare cells with sporadic immediate-early gene expression were commonly detected in PTCL. These findings have implications for the future development of EBV-targeting therapeutics for this cancer.

## 1. Introduction

Epstein-Bar virus (EBV) is a ubiquitous human herpesvirus with B-cell tropism that infects normal peripheral blood mononuclear cells (PBMC) [1]. Its causative association with the development of B-cells as well as epithelial malignancies, such as Burkitt lymphoma (BL), post-transplant lymphoproliferative disease (PTLD), Hodgkin lymphoma (HL), AIDS primary central nervous system lymphoma (CNS), nasopharyngeal carcinoma (NPC), and lymphoepithelioma-like carcinomas (LELC) of the gut and lung, is well established [1,2,3]. The constant etiologic role of EBV in the development of nasal NK/T-cell lymphoma (NKTCL) has also been confirmed in recent years [2,3,4]. However, the association of EBV with T-cell lymphoid malignancies is variable and less established [2,3,5].

Previously, we and others reported the detection of virus-harboring tumor cells in primary peripheral T-cell lymphomas (PTCL) in patients without overt preexisting immunodeficiency [2,6]. Intriguingly, in PTCL biopsies, a proportion of EBV-infected tumor cells presented with a loss of typical T-cell surface markers, along with the presence of some EBV-infected bystander B-cells. Moreover, reports have shown that there is an increase in tumor cell proportion along with PTCL tumor progression or recurrence, implying that the virus might facilitate the growth and survival of transformed T cells [7]. However, detailed descriptions of EB viral presence in PTCL biopsies, such as its pattern of gene expression at the molecular level and viral subtypes, are still scant [5,8]. 

The prototype patterns of lytic and latent infection by EBV were first defined from early studies of EBV infection and gene expression in B cells [9]. Many subsequent reports proved that such prototypes are generally useful to describe EBV gene expression patterns in both neoplastic and nonneoplastic cells of either B cell or non-B cell phenotypes [3,10,11,12]. Meanwhile, lytic EBV infection results in host cell lysis. In such an infection, all structural viral genes are expressed in the host cell, leading to the production and packaging of virions, final host cell lysis, and further spread of the virus. On the other hand, latent EBV infection allows the virus to persist in the host cell without cell lysis. In contrast to lytic infection, viral gene expression is quite restricted. Depending on the type of infected host cells, different viral genes may be restricted or expressed, and the patterns can be characterized as latency 0 to III. In brief, latency 0 is typically found in resting PBMC B-cells with the most restricted gene expression (only EBNA1 and BARTs expressed) [13,14]; latency I (EBNA1, BARTs, LMP2A expressed) is found in BL [15] and gastric cancer [16,17,18]; while latency II (EBNA1, BARTs, LMP1, LMP2A, 2B expressed) has been reported in multiple types of EBV-associated carcinomas and lymphomas [3,5,19]; and finally, latency III (Cp, all EBNAs, BARTs, LMP1, LMP2A, 2B expressed) is mostly found in B-cell malignancies in immunocompromised cases [1,2,3]. In primary nasal NKTCL, where EBV is 100% associated with the disease and present in almost every tumor cell, the viral gene expression pattern was found to be latency II [3,20]. However, it is also possible that the latency types are less definitively categorized in certain malignancies, especially because spontaneous lytic activation appears to be cell context-related [3,15,20], thus making it more intriguing to investigate the pattern of EBV gene expression in PTCL tumor biopsies [21].

While EBV infects certain neoplastic cell types, the patterns of viral gene expression in different neoplasms are apparently also affected by the cellular origin of the neoplasm. For example, as an epithelial neoplasm, NPC has strong LMP1 expression; yet gastric carcinoma is LMP1 negative [16,17,18]. In cases of EBV+ leiomyosarcoma in pediatric AIDS or organ transplantation populations, EBV expresses EBNA2 (from Cp) and LMP2A but not LMP1, indicating a unique latency [22,23]. PTCL collectively represents neoplasms originating from malignant lymphoproliferation of postthymic T cells [24]. Despite being classified by the WHO as mature T-cell and NK cell neoplasms [25], the prevalence rate of EBV infection in PTCL appears to be very different from that in NKTCL; hence, the EBV gene expression pattern and its regulation might also be different, demanding further investigation. Accumulating in vitro studies of EBV infection in T cell lines suggest that EBV gene expression in these malignant T cells might be both different from and more irregular than the typical patterns of expression reported in B cells. First, EBV-mediated oncogenic activities in T-cell lines are different than those in B lymphoblastoid cells [26]. Second, the pattern of viral gene expression in T-cells [27,28] also varies from that in B cells.

Patterns of EBV gene expression are controlled by specific EBV promoter activities. Therefore, while the gene expression pattern alone may provide essential clues about the latency types of the virus, a promoter activity study would give precise insights into the mechanisms of gene expression control. It is known that in latency 0, I, and II, a distinctive latent promoter Qp drives exclusive EBNA1 expression [15,29,30,31]. In latency III, two other latent promoters, Cp and Wp, are responsible for driving the expression of all six EBNAs [15,32,33]. On the other hand, Fp, located upstream of Qp, is shown to be an early lytic promoter [30,31,34]. Investigating EBV promoter usage and regulation in EBV + PTCL would add further insights into our understanding of the impact of viral infection in certain tumor microenvironments.

Overall, the mechanism of EBV involvement in the pathogenesis of PTCL is largely unclear. Intrigued by the results found in in vitro studies and to further clarify the patterns of EBV gene expression and its regulation in primary tissue biopsy samples, we comprehensively examined the viral gene and its protein expression patterns, viral promoter CpG methylation, and viral variants in EBV-infected primary PTCL biopsies.

## 2. Materials and Methods

### 2.1. PTCL Cases

Nine cases of EBV+ PTCL (Table 1) with sufficient snap frozen biopsy samples were selected from archival files of the Department of Pathology, Queen Mary Hospital, Hong Kong [35]. All cases were diagnosed according to the updated Kiel classification [36,37]. All cases had a rearranged TcRβ gene, thus being clonal in tumor clonality, except for case 5, which was not analyzed but had a rearranged TcRγ gene [35]. All cases were known to be EBV+ by EBER-in situ hybridization (ISH) or Southern blot hybridization for EBV clonality analysis [35]. EBER was detected in virtually all morphologically recognizable tumor cells in most cases; however, for cases 5, 7, 8, and t8, only a proportion of the atypical cells (~50%) were EBER+ [35,38]. Analysis of EBV clonality showed that the virus was clonal in three cases, biclonal in two cases, oligoclonal in one case, and lytic in one case (case 5) (Table 1). In some cases, double labelling [12] combined with immunostaining for the T-cell marker CD45RO (UCHL1) or B-cell marker CD20 followed by EBER-ISH was performed to confirm that the vast majority of EBER+ cells were of either the CD45R0+ or null phenotype [35], with scattered CD20+EBER+ reactive B-cells also detected.

### 2.2. RNA Extraction and RT-PCR

Total RNA was extracted from frozen tumor tissues and control cell lines by the acid guanidinum thiocyanate-phenol-chloroform method [39]. Using oligo-dT primers for reverse transcription, RT-PCR was carried out using a GeneAmp RNA PCR kit (Perkin Elmer Cetus, Norwalk, CT, USA) according to the company’s protocol [20]. The RT reaction product, equivalent to 0.5 µg of total RNA, was amplified in a 50 µL PCR volume for 40 cycles. Amplified products were electrophoresed on a 2% agarose gel, and the specificity was confirmed by Southern blot hybridization using ^32^P-end labeled internal oligonucleotide probes [20,31].

### 2.3. EBV-Specific Primers and Probes for RT-PCR

The analyzed EBV transcripts included EBNA1, EBNA2, LMP1, LMP2A, LMP2B, BamHI A rightward transcripts (BARTs), BZLF1 (Zta), BHRF1, and BLLF1. The usage of EBV C promoter (Cp), W promoter (Wp), Q promoter (Qp), and F promoter (Fp) was also examined [15,31]. The sequences of primers and internal oligonucleotide probes used followed previously published and verified ones: EBNA1 (QUK, YUK or FUK splice) [15,20,31,40], EBNA2 [5], LMP2A and 2B [40], BART [41], BZLF1 [13], BLLF1 [13], LMP1 [13,20], BHRF1 [20,34], and EBNA1 promoter usage [15,31].

### 2.4. DNA Extraction and EBV PCR Genotyping

DNA was extracted from snap-frozen tumor tissues and control cell lines as described previously [35]. Only six cases had sufficient tissue available for the PCR genotyping study. The primer combinations used and PCR conditions for EBNA3C and BamHI-F genotyping were as previously described [15,42], and the 30 bp deletion at the carboxyl terminus of the LMP1 gene was also as previously described [15,43]. For EBV genotyping, genome DNA samples were determined with PCR [15,43], using primers spanning EBNA3C, BamHI-F sites, and the LMP1 gene’s 30 bp deletion. PCR products were visualized either directly or detected after Southern blot hybridization.

### 2.5. Immunohistochemistry (IHC) for the Detection of EBV Proteins and Cell Surface Markers

IHC staining was performed on 6 µm cryostat sections for the detection of EBV LMP1 and Zta, using monoclonal antibodies (MoAb) CS.1-4 and BZ-1, respectively, and the standard alkaline phosphatase anti-alkaline phosphatase (APAAP) or streptavidin-biotinylated peroxidase complex (S-ABC) method (Dako, Kyoto, Japan), performed as previously described [12]. Cytospin slides of the Raji cell line were used as a positive control for LMP1 and the B95-8 cell line as a positive control for Zta immunostaining. Antigen retrieval using microwave irradiation pre-treatment in 0.01M Trisodium citrate solution (pH 6.0) was used for cell surface marker staining on paraffin sections for IHC combined with ISH. Normal serum was used as a negative control.

### 2.6. Promoter CpG Methylation Analysis of Bisulfite-Converted DNA

The CpG methylation status of EBV Cp and Wp was examined by methylation-specific PCR (MSP) on bisulfite-treated genomic DNA, using primers and conditions described previously [15,31,44,45,46]. Briefly, 25 ng of bisulfite-treated DNA was PCR amplified by 40 cycles in a 12.5 µL reaction volume with validated strand-specific primer pairs selectively targeting the methylated or unmethylated EBV DNA sequences of the Cp and Wp promoters [15,31,44,45,46] using a specific PCR system. PCR products were analyzed on a 2% agarose gel.

### 2.7. ISH for EBV-Encoded Small Nuclear RNA 1 and 2 (EBER) and BHLF1

ISH for EBER and BHLF1 RNA was carried out on 5 µm paraffin sections using the EBV-ISH kit from Dako (Kyoto, Japan) under RNase-free conditions [47], with fluorescein-conjugated EBER or BHLF1 oligonucleotide probes. Paraffin blocks of the EBV+ cell line B95-8 (lytic) were used as positive controls and EBV-negative B-cell line BJAB as negative control. RNase A-treated serial sections and a hybridization buffer without probe were also used as negative controls. Sections were counterstained with nuclear fast red. For double-staining combining IHC of cell surface markers with EBER-ISH, IHC followed by ISH was carried out as described previously [12].

### 2.8. Double Immunohistochemistry

Double IHC was performed on frozen sections by combining APAAP for CD surface markers and indirect immunofluorescence for LMP1, as previously described [47,48].

## 3. Results

### 3.1. Expression of EBV Latent, Immediate-Early, and Lytic Genes

**Figure 1:**
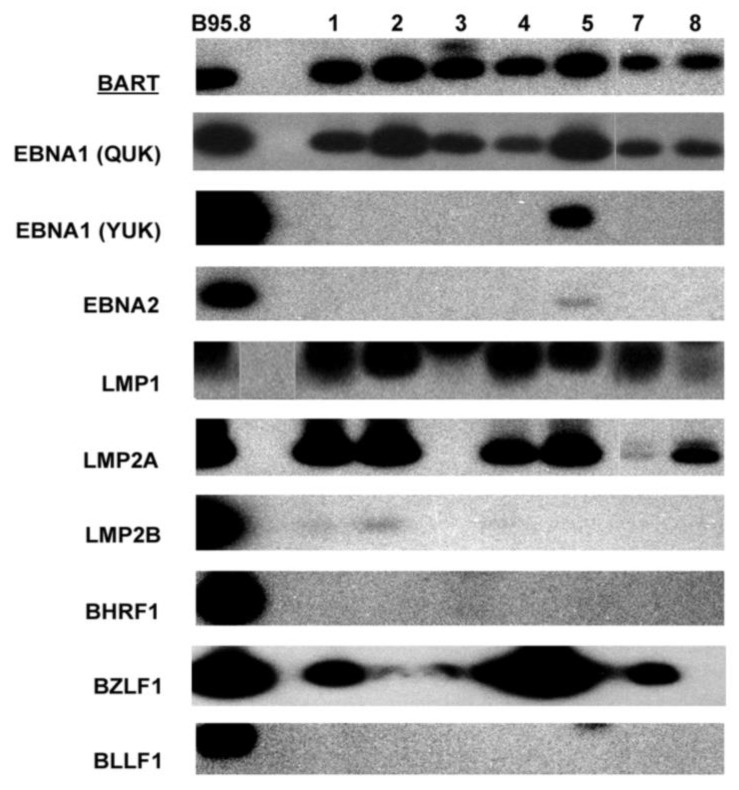
Autoradiograph images showing Southern blot hybridization of RT-PCR products for various EBV gene transcripts detected in seven PTCL cases. The B95-8 cell line was used as a positive control.

In total, nine PTCL cases with both frozen and paraffin tissue blocks were studied. Seven cases had RNA samples available for EBV gene expression examination by RT-PCR. Strong BART mRNA expression was detected in all cases, confirming the presence of EBV and validating the quality of the sample RNA used for the RT-PCR experiments (Figure 1, Table 1). Only case 5 expressed both QUK- and YUK- spliced EBNA1, with EBNA2 mRNA also being very weakly detected, whereas the other six cases all expressed only QUK-spliced EBNA1. LMP1 mRNA was detected in all cases, and LMP2A mRNA was only detectable in six cases, with varying quantities as reflected by the difference in band intensities. LMP2B mRNA could only be faintly detected in three cases.

**Figure 2:**
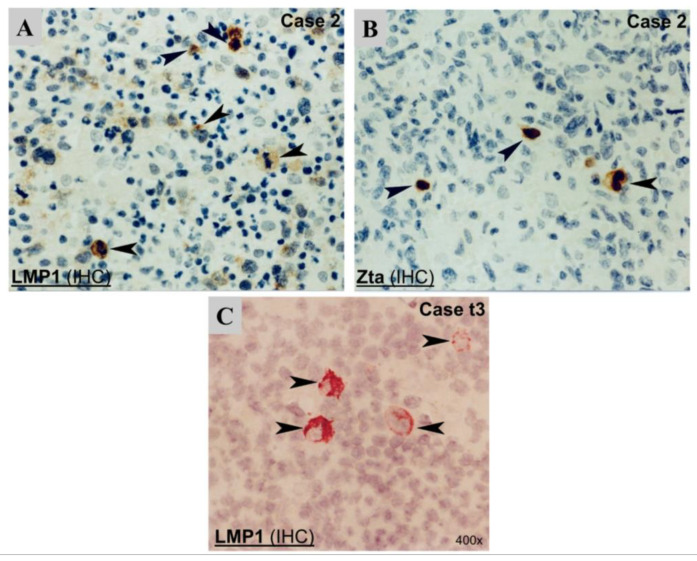
Detection of EBV protein expression by immunohistochemistry (IHC). (**A**) LMP1 protein shown by cytoplasmic staining in a pleomorphic medium-sized and large T-cell lymphoma case (case 2); (**B**) ZEBRA protein shown by strong nuclear staining in the same case. (**C**) LMP1 protein, shown by strong cytoplasmic staining in a case (case t3). Black arrowheads indicate EBV protein-positive cells.

By immunostaining, a few clustered LMP1+ cells were detected in 7/8 cases with available samples (Figure 2). In one case (t8), only rare isolated LMP1+ cells were detected. LMP1+ cells appeared to be isolated cells with enlarged morphology (Figure 2). Double IHC confirmed that the LMP1+ cells were of the T-cell phenotype (CD3+) (Table 1).

The expression levels of EBV immediate-early and lytic gene transcripts varied among cases (Figure 1, Table 1). Interestingly, mRNA expression of the immediate-early gene that is essential for lytic replication, BZLF1 (Zta), could be detected at varying levels by RT-PCR in 6 cases, with case 5 showing the strongest BZLF1 band (Figure 1). In agreement with these findings, rare scattered +ve cells for Zta were detected by immunostaining in 5/6 cases (Figure 2). However, no expression of late lytic genes (BHRF1, BLLF1) was detected by RT-PCR or immunostaining in any of the cases (Figure 1).

In summary, all cases were BART+EBNA1(QUK)+LMP1+, with the majority of cases also being LMP2A+BZLF1+. Case 5 differed from the others in that it had BART+EBNA1(QUK+YUK)+EBNA2+LMP1+LMP2A+BZLF1+ (Figure 1).

### 3.2. EBNA1 Promoter Usage and CpG Methylation Status

**Figure 3:**
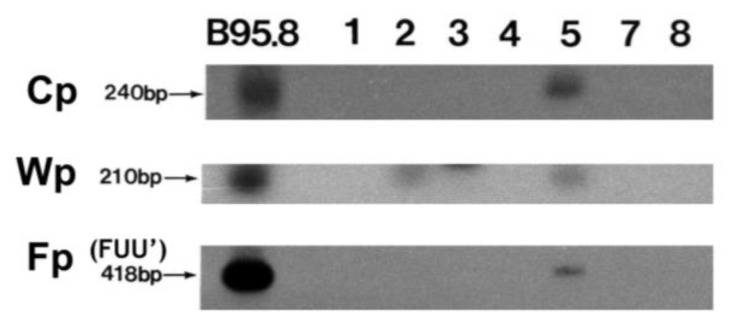
Promoter usage for EBNA1 expression. Autoradiograph showing Southern blot hybridization of RT-PCR products from EBV gene transcripts differentially initiated from Cp, Wp, and Fp promoters in seven PTCL cases. B95-8 was the positive control.

Analysis of EBNA1 promoter usage was performed by RT-PCR for promoter-specific expression and promoter CpG methylation analysis using bisulfite DNA. Predominant Qp usage (QUK) for EBNA1 was detected in all cases (Figure 1). In contrast, Cp, Wp, and Fp usages were mostly not detected, except for Cp usage (YUK, Cp) and a weak Wp usage signal in the exceptional case 5 (Figure 3, Table 1). In case 5, the lytic splice form (FUU’) of the lytic EBNA1 promoter Fp [15,31] was also weakly detected (Figure 3). Thus, case 5 has exceptional usage of the four EBNA1 promoters Cp, Wp, Qp, and Fp.

**Figure 4:**
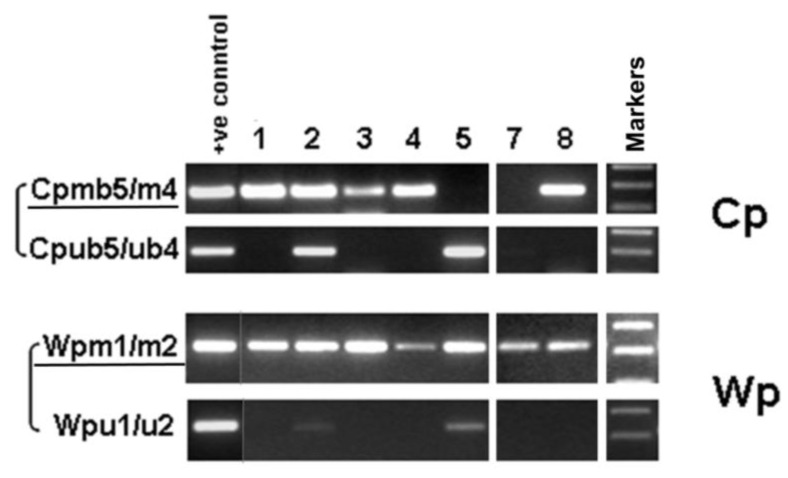
Analysis of the CpG methylation status of EBV Cp and Wp in PTCL by methylation-specific PCR (MSP) using bisulfite genomic DNA. “m” primers were used for methylated promoters, while “u” primers were used for unmethylated promoters.

Promoter CpG methylation analysis confirmed the RT-PCR results, showing that Wp was clearly methylated for all but the lytic case (case 5) which was weakly unmethylated (Figure 4). Cp was shown to be fully methylated in four cases, half methylated in one case (case 2), undetectable in one case, and fully unmethylated in case 5 (Figure 4). This lytic pattern of viral gene expression in case 5 correlated with its lytic EBV genome DNA pattern as detected in previous Southern blot analyses (Table 1) [35].

### 3.3. Detection of the Sporadic Expression of Early Lytic Genes

**Figure 5:**
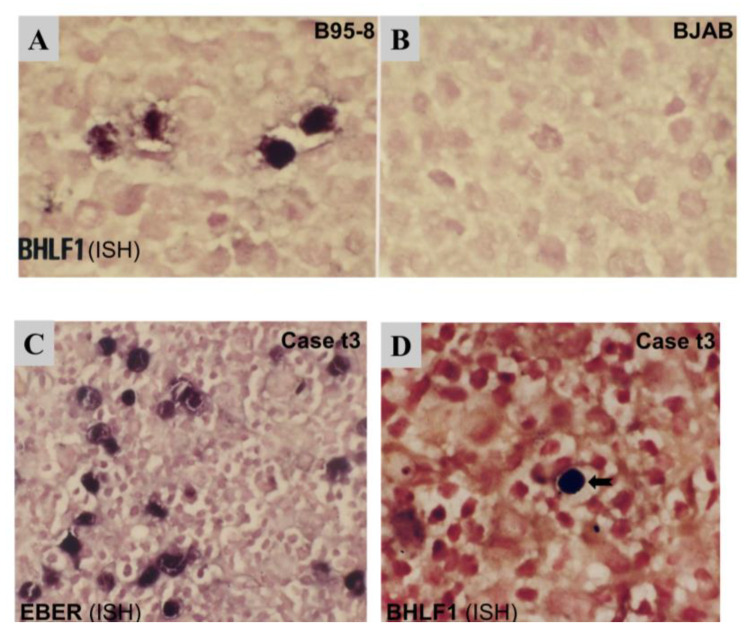
ISH for the detection of BHLF1 and EBER transcripts. (**A**) B95-8, an EBV+ lytic cell line, was used as a lytic positive control for BHLF1. (**B**) BJAB, an EBV-negative cell line, was used as a negative control. (**C**) A PTCL case (case t3) was shown to be EBV+ by being EBER+. The vast majority of tumor cells were EBER+ with a diffuse pattern of distribution. (**D**) The BHLF1 transcript was shown to be expressed in rare, isolated EBV+ cells in EBER+ case 3t.

As the expression of the immediate-early gene BZLF1 was detectable by RT-PCR in the majority of cases (Figure 1) and its coded protein Zta was also detected in rare cells in most cases by immunostaining (Figure 2), we sought to further examine the expression of early lytic gene BHLF1 by in situ hybridization (ISH) in two cases with additional paraffin tissue available, with EBV+ lytic cell line B95-8 and EBV-negative cell line BJAB as positive and negative controls, respectively. First, ISH results showed a diffuse or clustered pattern of EBER+ tumor cells in each case (Figure 5). EBER was detected in virtually all morphologically recognizable tumor cells in the diffuse case (t3) but in only ~50% of tumor cells in the clustered case (t8), which is similar to our previous report [35]. In the case (t3) with a diffuse pattern of EBER+ tumor cells, double labeling of EBER-ISH and IHC was performed, and the results showed that virtually all EBER+ cells were of the T-cell phenotype (CD45R0+). Interestingly, a rare isolated, single-cell pattern of BHLF1+ cells (<1 cell per 20x medium power field) was also detected in this case (t3) (Figure 5), indicating sporadic expression of the EBV early lytic gene in very rare cells. However, no expression of late lytic BLLF1 protein was detected by immunostaining in this case (Table 1).

### 3.4. Dominant Presence of Type-A EBV Infection

**Figure 6:**
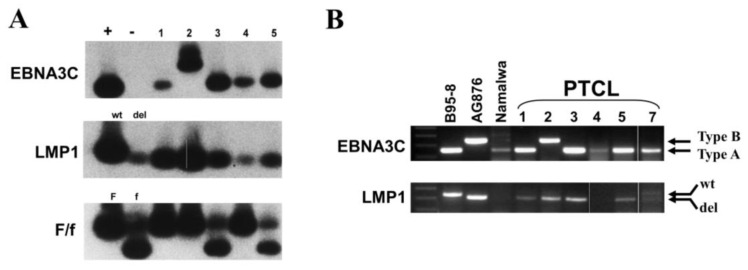
EBV genotyping of PTCL cases, including the EBNA3C variation, the LMP1 gene 30 bp deletion, and the BamHI F/f variation. (**A**) Autoradiograph images showing Southern blot hybridization of PCR products. (**B**) Agarose gel electrophoresis of PCR products from a separate assay. EBNA3C had a 153 bp product for type-A EBV and a 246 bp product for type-B EBV. A 30 bp deletion at the LMP1 carboxyl terminus is shown by the shorter PCR product of 286 bp rather than a 316 bp band for the wild-type gene. BamHI F is shown by an uncut PCR product band of 222 bp, whereas the BamHI f gene is shown by a cut PCR product with two bands of 125 bp and 97 bp. The controls used are as follows: for the EBNA3C gene, the B95-8 cell line is type-A, and the AG876 cell line is type-B. For the LMP1 gene, B95-8 is wild-type, and Jijoye or AG876 is deletion-type. For BamHI F/f variation, B95-8 is the positive control for BamHI F and Jijoye for BamHI f.

Genotype analysis of EBV by PCR revealed that the majority of the cases carried type-A EBV, with only case 2 bearing type-B EBV (Figure 6). The 30 bp deletion of the LMP1 gene was present in all of the cases tested. There was no apparent dominance of either the F or f variant in the examined cases (Figure 6A; Table 1).

## 4. Discussion

In this study, we comprehensively examined EBV gene expression and its regulation in PTCL primary tumors, although with a modestly small case number. All cases detected were BART+EBNA1(Qp)+LMP1+, with the majority also being LMP2A+ BZLF1+. Thus, this pattern of EBV gene expression in PTCL (BART+EBNA1(Qp)+LMP1+LMP2A+BZLF1+) is the same as the typical latency II pattern, as previously reported in EBV-associated B-cell and NK/T cell lymphomas (HL, NKTCL) [20,41] and carcinomas (NPC) [15,19,31,40], and comparable with earlier reports for PTCL tumors [5,8]. Moreover, sporadic expression of the EBV early lytic gene in very rare cells could also be detected in some cases.

The EBV latency types were essentially established from early studies performed in EBV-infected B lymphoblastoid cells [9,49]. It has since been validated in multiple studies in later years that EBV gene expression patterns follow unique regulatory programs according to the lineage of its host cells. Some in vitro studies of EBV infection in T-cells demonstrated that EBV may act as an oncogenic factor in T-cells in a way that is distinct from that found in B lymphoblastoid cells [26]. Specifically, a study of EBV gene expression in an infected MT-2 T-cell line revealed that, in addition to the typical latency II pattern, Cp/Wp activities and early lytic gene expression were also detected [27]. In contrast to that found in MT-2 T-cells, where both Cp/Wp and Qp were active, EBV promoter gene expression in our PTCL biopsies was mostly restricted to Qp only, with the absence of Cp and Wp transcripts. This was further confirmed by promoter CpG methylation analysis, which showed that Cp and Wp were largely methylated and thus silent. The overall predominant utilization of Qp, without activation of Wp, Cp, or Fp, clearly indicated a restricted EBV gene expression program/latency, similar to that seen in viral latent persistence in other lymphoid (HL, NKTCL) [20,50,51] and epithelial tumors (NPC) [15,19,31,40] and distinct from malignant B-cells in BL and immunocompromised situations [15]. Thus, our promoter CpG methylation analysis confirms the latency II pattern of EBV infection at the gene regulatory level in PTCL cases.

However, Chen et al. [5] only detected LMP1 protein in one of their six cases, whereas our IHC staining revealed LMP1 protein in all eight cases examined, despite the positive staining being limited to a few isolated medium to large sized atypical cells of CD3+, or null phenotype (Table 1, Figure 2). Another study also detected LMP1 protein by immunostaining in all cases [8]. Similar to those demonstrated in EBV oncogenic capacity in B-cells [52], a functional study by Takada et al. [53] revealed that in vitro EBV infection of the EBV-negative T-cell line MOLT4 also displayed a latency II pattern. Moreover, the expression of the viral protein LMP1 has been shown to directly induce NF-κB activation in MOLT4 cells [53]. In this context, our speculation about the major pathogenic implications of our frequently observed LMP1 expression in PTCL biopsies is strongly supported.

Some previous studies of EBV infection in T-cells suggested the possibility that they are relatively more permissive for the expression of viral immediate–early genes than EBV-infected B-cells [26,27]. Similar to that seen in MT-2 T cells [28], we detected frequent mRNA expression of an immediate-early gene, BZLF1, in our PTCL cases. By IHC staining, there was also sporadic detection of Zta, the BZLF1-encoded protein, in rare isolated cells in some of our cases. Interestingly, BZLF1 expression in T-cells was demonstrated in at least two other in vitro studies [26,54] and was also commonly detected in NKTCL [20]. The detection of Zta in rare isolated cells in some PTCL cases has also been reported previously [5,8]. This type of abortive activation of EBV replication has been reported in rare Hodgkin and Reed-Sternberg cells of HL [55]. However, it is worth noting that, in EBV-associated epithelial tumors (NPC and gastric cancer), Zta is rarely (or not at all) detected by immunostaining or RT-PCR [16,17,18,19], reflecting a possible cell type-context issue.

Although the immediate–early gene proteins Zta and Rta are essential for the lytic replication of EBV, the lack of expression of late lytic genes such as BLLF1 (MA) and the absence of infectious virion particles indicate that lytic activation is abortive, as in the case of EBV+ HL [55]. Thus, it is reasonable to hypothesize that these tumor cells do not enter into productive viral infection unless exceptional conditions exist. On the other hand, the expression of Zta protein might have more pathogenic implications than its role in lytic activation. On this point, Zta protein has been shown to be associated with altered p53-dependent transcription during malignant transformation of T-cells in in vitro studies [54]. Thus, the detection of Zta in PTCL biopsies may infer its pathogenic role during the transformation toward T cell malignancy.

One exceptional case (case 5) had lytic EBV infection and a full EBV gene expression pattern (BART+EBNA1(Qp+Cp)+EBNA2+LMP1+LMP2A+BZLF1+). This lytic pattern of viral gene expression correlated with its lytically replicating viral DNA, as previously detected by Southern blot analysis [35]. This pattern was further supported by the detection of lytic promoter Fp activity and EBNA2 mRNA expression, indicating that the dominant presence of lytic EBV infection in tumor cells in this case was likely leaking from a latency III-type infection. This type of lytic EBV infection together with a full latency III expression pattern has also been recently detected in a single PTCL case of a distinct anaplastic large-cell lymphoma subtype [56]. For case 5, apart from its strong association with EBV, its histology type—angioimmunoblastic T-cell lymphoma (AIL)—is also known for its display of oligoclonal B cell proliferation [24]. Recent investigations of EBV infection in AIL by Nakhoul et al. [56] and others with in vitro studies [56,57,58], support the possibility that EBV+ B cell infiltrates also play a role in immune modulation in the tumor microenvironment. As EBV+ B cell infiltrates are commonly detected in some subtypes of PTCL [35,59], such evidence also favors the pathogenic impact of EBV+ B cell infiltrates.

EBV has been shown to downregulate the expression of host cell surface markers or differentiation markers [60], possibly to evade immune recognition and promote host tumor cell growth/survival. While the tumor cells in our previous study of EBV+ PTCL were of the CD4+CD8-CD56- phenotype, we found that various proportions of EBER+ cells were of the null type (CD45RO-CD3-CD4-, also CD20-) [35]. It is plausible to speculate that the null phenotype feature may be attributed to EBV infection-induced downregulation of cell surface markers. However, in case t3 of this study, where double labeling was performed, virtually all EBER+ cells were of the T-cell phenotype (CD45R0+), indicating the complexity of PTCL in terms of EBV infection and phenotypes. In some PTCL cases, EBV might be detected (by EBER-ISH) in only a proportion of morphologically recognizable tumor cells [35], which might reflect a parallel hit-and-run phenomenon as suggested in the situation of HL [61,62].

Previous studies suggest that the type-A EBV strain may possess more potent transforming ability in certain cell lines, and that the 30 bp deleted type of LMP1 gene may be a more potent transforming factor [56,58]. All of our cases, except for one, harbored type-A EBV, and all cases had the LMP1 gene with a 30 bp deletion, thus strengthening the potential oncogenic roles of these EBV variants in tissue environments and our locality.

In summary, the overall findings of our study showed correlations with the in vitro study results of EBV infection in T cell lines. In conclusion, most of our PTCL cases showed a latency II pattern of EBV gene expression similar to HL, NKTCL, and NPC, in addition to the expression of the early lytic gene BZLF1 mRNA and its protein Zta in rare EBV+ cells in most cases. Moreover, rare PTCL cases with uncommon latency III combined with lytic activation could also be detected. These findings support the hypothesis that EBV plays an active role in T-cell tumorigenesis in EBV+ PTCL, which has implications for the future development of EBV-targeting therapeutics for this cancer.

## Figures and Tables

**Table 1:** Summary of EBV immunostaining, gene expression, promoter usage and genotyping data of PTCL.

				IHC		RT-PCR and MSP															PCR Genotyping		
									MSP			EBNA1				LMP2							
Case No.	Subtype	Biopsy Site	Clonality (EBV)	LMP1	Zta	BART	Cp	Wp	Cp methyl	Wp methyl	Fp (FUU’)	QUK	YUK	EBNA2	LMP1	2A	2B	BZLF1	BHRF1	BLLF1	LMP1 gene	EBNA 3C	BamHI F/f
**1**	AIL	LN	Clonal	**+**	**+/-**	**++**	**-**	**-**	**m**	**m**	**-**	**++**	**-**	**-**	**++**	**++**	**+/-**	**+**	**-**	**-**	del	A	F
**2**	PL	LN	Clonal	**+**	**+/-**	**++**	**-**	**-**	**m + (u)**	**m**	**-**	**++**	**-**	**-**	**++**	**++**	**+/-**	**+/-**	**-**	**-**	del	B	F
**3**	MF	Skin	Biclonal	**+**	**-**	**++**	**-**	**-**	**m**	**m**	**-**	**++**	**-**	**-**	**+/-**	**-**	**-**	**+/-**	**-**	**-**	del	A	f
**4**	Unc	LN	Clonal	*ND*	*ND*	**++**	**-**	**-**	**m**	**m**	**-**	**++**	**-**	**-**	**++**	**++**	**+/-**	**+**	**-**	**-**	del	A	F
**5**	AIL	LN	Lytic	**+**	**+/-**	**++**	**++**	**+**	**u**	**m + (u)**	**+**	**++**	**+**	**+/-**	**++**	**++**	**-**	**++**	**-**	**-**	del	A	f
**7**	AIL	LN	Biclonal	**+**	**+/-**	**++**	**-**	**-**	*nil*	**m**	**-**	**++**	**-**	**-**	**++**	**+/-**	**-**	**+**	**-**	**-**	wt + del	A	
**8**	LeL	LN	Oligoclonal	**+**	**+/-**	**++**	**-**	**-**	**m**	**m**	**-**	**++**	**-**	**-**	**+/-**	**+**	**-**	**-**	**-**	**-**			
**t3**		LN	(TcRβ-R)	**+@**	**+/-**	EBER**++**$ (ISH)								**-** (IHC)					**+/-** (BHLF1-ISH)	**-** (IHC)			
**t8**		LN	(TcRβ-R)	**+/-**	**-**	EBER**+** (ISH)								**-** (IHC)					**-** (BHLF)	**-** (IHC)			
B95-8 (+ve control for EBV expression)				**+**	**+**	**++**	**++**	**+**			**+**	**++**	**++**	**++**	**++**	**++**	**++**	**++**	**+/-**	**++**	wt	A	F
Molt4 (**-**ve control)						**-**	**-**	**-**			**-**	**-**	**-**	**-**	**-**	**-**	**-**	**-**	**-**	**-**			
Jijoye (+ve control for f variant)																					del	-	f

Subtype (updated Kiel): AIL: angioimmunoblastic T-cell lymphoma; PL: pleomorphic medium-sized and large T-cell lymphoma; MF: mycosis fungoides; unc: PTCL, unclassified; LeL: lymphoepithelioid lymphoma; LN: lymph node; TcRβ-R: TcRβ gene rearranged. nil: not detected. For RT-PCR analysis: ++: strongly positive, +: positive, +/-: weakly positive, -: not detected; For ISH analysis: ++: almost all atypical tumor cells positive, +: ~50% tumor cells positive, +/-: only rare isolated cells positive; $: virtually all the EBER+ cells were confirmed of T-cell (CD45R0+) phenotype. For IHC: +: a few clustered positive cells, +/-: only rare isolated positive cells; -: not detected. @: LMP1+ cells were confirmed to be of T-cell phenotype (CD3+). For PCR genotyping: wt: wild-type LMP1 gene (B95-8 prototype); del: deleted type LMP1 gene with a 30-bp deletion at its carboxyl terminus; A: A-type EBV; B: B-type EBV; F: absence of a BamHI F fragment of the EBV genome; f: presence of an extra BamHI site in the BamHI F fragment of the EBV genome.

## Data Availability

Not applicable.
